# Plaies des membres par agression: analyse de 245 dossiers

**DOI:** 10.11604/pamj.2015.22.183.4434

**Published:** 2015-10-23

**Authors:** Monsef Boufettal, Mustapha Mahfoud, Farid Ismael, Mohamed Kharmaz, Ahmed El Bardouni, Mohamed Saleh Berrada, Moradh El Yaacoubi

**Affiliations:** 1 Clinique Universitaire de Traumatologie Orthopédie, Centre Hospitalier Universitaire Avicenne, Faculté de Médecine, Université Mohammed V, Rabat, Maroc

**Keywords:** Plaies, membres, agressions, épidémiologie, wounds, limbs, assault, epidemiology

## Abstract

Il s'agit d'une étuderétrospective, analytique, monocentrique rentrant dans le cadre d'une étude épidémiologique s’étalant sur une période de trois années (de 2010 à 2012) durant laquelle nous avons revu les dossiers de 245 patients victimes de violence et d'agression. Nous avons exclu les lésions simples traitées en ambulatoire. Par conséquent, nous nous sommes limités aux cas de blessures ayant nécessité une prise en charge spécialisée au bloc opératoire. Les objectifs de notre travail étaient de connaitre la fréquence des agressions au service de traumatologie du CHU de Rabat, classer les différents types de lésions, évaluer leur gravité, mettre la lumière sur les populations les plus touchées et enfin montrer les différentes modalités de prise en charge thérapeutiques.

## Introduction

La croissance démographique des grands centres urbains au Maroc est à l'origine de l'augmentation des problèmesde Santé qui caractérisent les grandes villes. Ces problèmes, notamment les accidents routiers et les agressions, constituent une préoccupation importante de santé publique. Le centre hospitalier universitaire de Rabat participe à l'accueil des victimes. La présenteétudeporte sur 245 dossiers de patients victimes d'agressions admis aux urgences traumatologiques durant une période de trois ans. Elle rapporte les circonstances étiologiques, les lésionsrencontrées et leur gravité, décrit leur prise en charge, et permet une analyse global des caractéristiques épidémiologiques des plaies des membres recueillies.

## Méthodes

Il s'agit d'une étude épidémiologique qui s'est intéressée à l'analyse rétrospective de 245 dossiers de patients présentant des plaies secondaires à des agressions physiques sur une durée de trois ans. Les critères d'inclusion étaient le motif principal de consultation qui est « la plaie ». Nous avons exclu les lésions simples traitées en ambulatoire ainsi que les patients présentant des plaies multiples (abdominales, thoraciques, ou cardiaques) ayant nécessité une prise en charge pluridisciplinaire. Par conséquent, nous nous sommes limités aux cas de blessures localisées aux niveaux des membres ayant nécessité une prise en charge spécialisée au bloc opératoire. Notre fiche d'exploitation comportait des éléments a visée épidémiologiques (l’âge, le sexe, la profession, la date, le lieu et le mode d'agression de la victime), cliniques (le type de lésions, leurs localisations anatomiques et les conséquences lésionnelles) et thérapeutiques (le traitement reçu, les résultats fonctionnelles et les complications).

## Résultats

**Figure 1:**
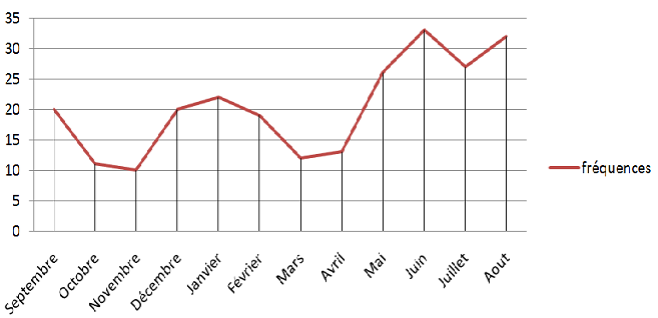
Évolution de la fréquence des agressions selon les mois pour les 3 années

245 victimes de violence par agression ont nécessité une prise en charge spécialisée et une hospitalisation au service de traumatologieet orthopédie. Ce fléau touche essentiellement une population jeune avec un âge moyen de 25ansavec des extrêmes allant de 17 à 70ans avec une nette prédominance masculine 75,51% de l'ensemble des patients étudiés (Tableau 1). Nous constatons une concentration des agressions à Rabat avec 115 cas, contre 95 cas en provenance de Salé et 37 cas en provenance de Témara. L'importance de ce phénomène a été enregistrée essentiellement durant les jours du week-end et plus particulièrement le samedi,avec une augmentation significative en été, spécialement au mois de juin avec le pourcentage le plus élevé de l'année qui est de 13.47% (Figure 1). Différents types d'armes sont utilisés, à savoir: les armes blanches, les bâtons et les morceaux de verre. Dans notre série, l'arme blanche reste le moyen d'agression le plus utilisée et retrouvée dans 152 cas. Les lésions intéressaient aussi bien le membre supérieur que le membre inférieur avec une atteinte prédélective au niveau de l'avant-bras (Tableau 2). Sur tous les patients étudiés, 43% se sont présentés avec des lésions complexes comportant une association des lésions musculo-tendineuses, osseuses, nerveuses et /ou vasculaires. Pour ce qui est des lésions isolées, l'atteinte musculo-tendineuse reste la plus importante avec un taux de 29% (Tableau 3). Le délai de consultation était compris entre 01 heure et 36 heures avec une moyenne de 08 heures. Tous les patients de notre série ont bénéficié d'un examen clinique systématique et global dès leur admission aux urgences permettant de rechercher d'autres localisations ou l'atteinte d'organes vitaux. Le bilan para clinique était systématique et guidé par l'examen clinique, suivi, en fonction des points d'appel, d'examens plus spécifiques.

**Table 1:** Nombre d'agressions par année et par sexe

	Nombre d'agressions par année		
	2010	2011	2012
Homme	56	74	82
Femme	6	11	16

**Table 2:** Répartition des lésions selon le membre lésé pour les 3 années

			Effectifs	Fréquences	Total
Membre supérieur	Distal	Main gauche	28	11,43%	**57.93%**
		Main droite	11	3,49%	
		Poignet gauche	10	3,03%	
		Poignet droit	1	0,40%	
		Avant-bras gauche	74	30,20%	
		Avant-bras droit	23	9,38%	
	Proximal	Coude gauche	12	4,89%	**24.68%**
		coude droit	10	4,08%	
		bras gauche	30	11,24%	
		bras droit	3	1,22%	
		épaule gauche	6	2,44%	
		épaule droite	2	0,81%	
Membre inférieur	Distal	Jambe gauche	11	3,49%	**6.75%**
		jambe droite	8	3,26%	
	Proximal	Cuisse gauche	17	5,93%	**10.64%**
		cuisse droite	14	4,71%	

**Table 3:** Type de lésions causées par les agressions avec leurs fréquences pour les 3 années

Type de lésion	Fréquences
Musculaire et tendineuse	29%
nerveuse	4%
Osseuse	22%
Vasculaire	2%
lésions complexes	43%

Tous nos patients ont bénéficié d'un bilan biologique standard pré-anesthésique à savoir: numération formule sanguine, Ionogramme sanguin, TP-TCA ainsi qu'un bilan radiologique comportant une radiographie du membre lésé de face et de profil. Une radiographie pulmonaire a été demandée pour la majorité de nos patients, dans le cadre d'un bilan pré-anesthésique. La majorité des patients ont été pris en charge dans un délai de 12 à 24 heures et environ un tiers au-delà de 24h. La variation du délai de prise en charge est liée principalement à la disponibilité du bloc opératoire et à l'importance du flux des traumatismes gravesprioritaires au bloc opératoire. Pour tous les patients, un traitement médical a été administré, à savoir: une sérovaccination anti-tétanique, une antibiothérapie prophylactique (si besoin), une analgésie à base d'anti-inflammatoires et d'antalgiques palier 1 et 2. Le traitement chirurgical dépendait du bilan lésionnel et consistait en une réparation tissulaire associée à une fixation osseuse (si nécessaire). Sur les 3 années étudiées, la durée moyenne d'hospitalisation était entre 2 à 3j par patient. En général, les résultats fonctionnels étaient bons pour les lésions osseuses et musculo tendineuses tandis que pour les lésions nerveuses et les lésions complexes les résultats étaient moyens voir mauvais (Tableau 4). Ces résultats ont été évalués pour chaque groupe lésionnel selon plusieurs critères: la douleur évaluée par l’échelle visuelle analogique (EVA); la mobilité; la force musculaire; la récupération sensitivomotrice; le degré d'impotence fonctionnelle; le retentissement socioprofessionnel.

**Table 4:** Résultats fonctionnels selon les lésions

Lésions	Bon	Moyen	Mauvais
Osseuses	95%	5%	0%
Musculo-Tendineuses	75%	15%	10%
Nerveuses	40%	43%	17%
Vasculaires	61%	-	39%
Lésions complexes	28%	32%	40%

## Discussion

Les plaies représentent environ 13% des admissions au service des urgences, les situant dans les tous premiers rangs des motifs de recours, et intéressent principalement la tête, le membre supérieur, et le membre inférieur [1]. Dans notre pays les violences par agression constituent un mode de recrutement de plus en plus fréquent en traumatologie [2]. Les lésions liées aux agressions se caractérisent par leurs polymorphismes et leur gravité très variable. La prise en charge initiale d'une plaie répond donc à des exigences extrêmement variables tenant compte du terrain, de la localisation de la blessure et de son mécanisme. Sa qualité conditionne pour une large part l’évolution ultérieure [1].

La proportion des hommes victimes est prédominante correspondant à des violences urbaines contrairement aux femmes majoritairement victimes de violences domestiques [3]. Ceci répond aux résultats de notre série et pourra être expliqué par la fréquence des sorties nocturnes, la consommation d'alcool et des drogues souvent retrouvées chez la population jeune masculinetandis que les femmes sont plus concernées par les violences conjugales. Ceci reflète l'importance de ce phénomène durant les weekends et les périodes d’été ou la clémence climatique, les vacances et le changement du rythme citadin favorisent les sorties diurne et nocturne. L'agresseur a souvent recours à plusieurs agents vulnérants de nature très variable. En France, les plaies par armes blanches représentent la cause la plus fréquente de plaies pénétrantes et surviennent principalement au décours d'une agression [4].

Notre étude rejoint la majorité des séries qui confirme la prédominance des lésions au niveau du membre supérieur [2, 5]. Ceci pourrait être expliqué d'une part, par le fait que le membre supérieur est souvent visé par l'agresseur (vols de bijoux, téléphone portable…) et d'autre part, par le fait qu'il sert souvent de moyen de défense pour la victime. Les lésions complexes restent les plus fréquentes dans notre série ainsi que dans la série de POTARD et ASSALIT [6, 7], ceci pourrait s'expliquer en partie par la complexité anatomique du membre supérieur ainsi que la proximité des éléments anatomiques pouvant engendrer non seulement des lésions simples mais aussi des lésions de voisinage.

L’évaluation du patient nécessite un examen clinique rigoureux et des connaissances anatomiques précises pour apprécier les risques selon la topographie de la plaie. Les décisions d'examens complémentaires et les actions thérapeutiques sont prises de façon collégiale entre urgentistes, chirurgiens, réanimateurs et radiologues [4]. Il faut comprendre que toute décision thérapeutique doit tenir compte de trois principaux facteurs [8]: la lésion: elle peut être, à elle seule, une indication d'un bloc opératoire lorsqu'il existe une atteinte profonde. La superficie et la localisation anatomique sont aussi des critères intervenant dans la décision; le contexte des urgences: le service des urgences est un lieu septique, ne disposant pas toujoursdu matériel, des compétences et du nombre de soignants nécessaires à l'anesthésie et au geste lui-même; le patient: le stress du patient, son terrain (conditions d'asepsie chez un patient immunodéprimé, par exemple) sont aussi des critères guidant la décision

L'agression physique est toujours vécue par les victimes et leur entourage comme le produit de diverses expériences. Cet acte ne reste pas sans conséquences pour la personne, on trouve des conséquences fonctionnelles (rétraction tendineuse, névrome, ischémie, raideurs, algodystrophie...) et psychologiques (dépression, angoisse, phobie, état de stress permanant, trouble du sommeil, trouble de l'humeur) [9, 10]. Cet état des lieux a permis une prise de conscience sur la nécessité de mettre en place des mesures préventives et des mesures d'amélioration de la prise en charge des patients victimes d'agression.

**La prévention:** elle repose sur plusieurs points: sensibiliser les enfants dès leur plus jeune âge dans les établissements scolaires contre les comportements agressifs; les médias devront éliminer la diffusion de portraits exprimant la violence; instaurer des mesures fermes pour limiter la consommation d'alcool et de stupéfiants; mesures préventives au cours de tout événement ou rassemblement public; amélioration des conditions socio-économiques, en particulier dans les banlieues; renforcement de la sécurité et des lois contre toutes sortes de violences; la prise en charge psychiatrique des agresseurs pour éviter les récidives.

**Les mesures d'amélioration:** mettre en place un circuit spécial aux urgences avec une structure d'accueil pour les personnes victimes d'agressions; prévoir une unité spéciale pour l'hospitalisation; formation des équipes médicales et paramédicales dans la prise en charge de ce type de malades; des conduites à tenir pratiques doivent être mises en place afin de permettre une prise en charge adéquate devant des plaies qui peuvent associer des lésions complexes des tissus mous, des lésions ostéo-articulaires ou vasculaires; coordination entre médecins des urgences, traumatologues, psychiatre, psychologue et l'unité de consultation médico-légale.

## Conclusion

Les agressions ne sont qu'une triste réalité, exprimant une colère, une violence de la part d'un adulte jeune. Ces agressions sont aujourd'hui présentes dans toute société et présentent des conséquences souvent redoutables. Nos résultats ainsi que la rareté des études médico-légales marocaines et à l’échelle internationale devraient inciter à poursuivre les travaux épidémiologiques dans les hôpitaux pour évaluer l’évolution de ce phénomène. De tels travaux soulignent également l'importance de l'unité de consultation médico-légale en complément des services d'urgences hospitaliers polyvalents au sein desquels le personnel devrait être spécifiquement formé à la prise en charge des victimes d'agression.

## References

[1] APNET, SAMU (2005). “Prise en charge des plaies aux Urgences. 12e conférence de consensus.”.

[2] Bouchra Bouyousfi (2001). Violence en traumatologie dans la ville de Marrakech.

[3] Lançon V, Moiron L (2009). “Victimes d'agressions: profils de consultation et limites des modalités actuelles de prise en charge dans un service d'accueil des urgences.”. Journal Européen des Urgences..

[4] Bège T, Berdah SV, Brunet C (2013). “Les plaies par arme blanche et leur prise en charge aux urgences.”. La Presse Médicale..

[5] Vion B (2010). “Prise en charge des plaies aux urgences étude de pratique au CHU d'Angers.”. Journal Européen des Urgences..

[6] Potard D, Petit G (1992). “La consultation des victimes de coups et blessures volontaires de l'Hôtel-Dieu de Clermont-Ferrand: bilan d'activité 1986-1990.”.

[7] Claude Assalit (1985). Bilan de deux années de consultations pour coups et blessures volontaires au chu de toulouse-rangueil.

[8] Hinglais E, Prével M, Coudert B (2005). “Plaies aux urgences, prise en charge.”. EMC-Médecine..

[9] Essalki Issam (2001). La violence en traumatologie (A propos de 522 cas).

[10] Chariot Patrick (2012). “Examen médical des personnes victimes de violence: fréquence des facteurs aggravants au sens du Code pénal, hétérogénéité des pratiques.”. La Presse Médicale..

